# Environmental factors associated with talent identification of women’s youth national team soccer players in the United States

**DOI:** 10.1371/journal.pone.0333065

**Published:** 2025-10-09

**Authors:** Matthew Andrew, Samuel Wood, José M. Oliva-Lozano, Mirelle Van Rijbroek, Rick Cost, Matthew J. Reeves

**Affiliations:** 1 Department of Sport and Exercise Sciences, Manchester Metropolitan University Institute of Sport, Manchester, United Kingdom; 2 Research and Innovation Department, United States of America Soccer Federation, Chicago, United States of America; 3 Bay Football Club, San Francisco Bay, United States of America; 4 Centre for Applied Sport, Physical Activity, and Performance, University of Central Lancashire, Preston, United Kingdom; 5 Department of Physical Sport Sciences, College of Sport Sciences & Physical Activity, Princess Nourah bint Abdulrahman University, Riyadh, Saudi Arabia; Portugal Football School, Portuguese Football Federation, PORTUGAL

## Abstract

Female soccer has seen an exponential growth in popularity leading to significant investment in talent identification and development processes. Soccer federations can only identify, develop, and select from a pool of players that are born within the country, thus environmental factors play an important role in the process. Despite the United States (US) being one of the most successful female soccer nations, little is known about environmental factors and their influence upon players’ talent pathways. This study sought to examine the key environmental factors associated with the identification and development of players for the Women’s Youth National Team (WYNT). Semi-structured interviews were undertaken with 23 experienced scouts (11.6 ± 8.8 yrs) that ranged from 32.0 to 61.8 minutes in length (46.1 ± 8.3 mins) to explore their perceptions of the female soccer landscape in the US and how different environmental factors can impact talent identification and development. Four higher order themes emerged: (1) no established playing style; (2) finding players for WYNT; (3) players access to talent opportunities; and (4) limited involvement of the Soccer Federation in youth development. This study is the first to examine these issues in female soccer and US contexts, and further examination of the association between environmental variables and talent identification and development are required to ensure evidence-based decision making that is both female- and country-specific.

## Introduction

In recent years, female soccer has seen an exponential growth in popularity [1], evidenced by participation rates, attendances, and funding. For example, female participation rates globally increased by 24% to 16.6 million between 2019 and 2023 (Fédération Internationale de Football Association (FIFA), [1]), with FIFA committing to increasing female soccer participation to 60 million globally by 2026 ( [2]). Indeed, many soccer federations (SF) are investing significant resources (e.g., personnel, finances, time) to professionalise their talent identification (TI) and development (TD) processes [3]. This is important at an international level, as SFs are only able to identify, develop, and select from a pool of players that are born (and/or their parents/grandparents) in the country. Despite the increased growth and investment in female soccer, currently there remains a lack of research [4,5], particularly studies focussed on examining TI and TD in female soccer [6,7].

Reviews of the literature (e.g., [8,9]) have identified that the TI and TD processes can be facilitated or attenuated by: (1) *the performer*, such as birthdate (e.g., relative age effect; [10,11]), genetics [12,13], anthropometrical, physical [14,15], and psychological skills (e.g., competitiveness, [16,17]); (2) *the environment* (e.g., birthplace; [18,19]), socio-economic status [20,21], and family support [22,23]; and (3) *practice*, such as sport-specific and other-sport practice [24,25], and pathways [26,27]. The environmental factors are particularly important as they are out of the control of the SF [28]. Moreover, each individual SF differs based on their geographic size (i.e., land mass), participation rates, finances, logistics, facilities, and depth of competition [29]. These variances have been suggested to underpin inconsistencies within the female soccer literature (e.g., relative age effect; [30,31]). Examinations of single SFs and their ‘soccer landscapes’ may reveal direct impact of the environment on their TI and TD processes [32–34]

The United States (US) is the fourth largest country by land mass, third most populous, and has the richest economy in the world. The female national teams are some of the most successful at youth (3 FIFA World Cups) and senior levels (4 FIFA World Cups; 5 Olympic Gold Medals; [35]). Soccer is one of the most popular US female sports, with ~1.52 million registered youth (<18 years) players [2]. It’s continued popularity and growth are demonstrated though the creation of a relatively new professional leagues (National Women’s Soccer League (NWSL)) as well as increased attendances, both live and televised, at recent domestic and international tournaments [36]. Whilst the US soccer environment shares similarities with other SFs, they also have divergent features within their TI and TD processes. For instance, multiple unique pathways can be followed for players to reach professional status in the US. Most players begin playing soccer at a regional level during youth (4–11 years; [27]), before progressing to national level competition platforms (e.g., Girls Academy (GA), Elite Clubs National League (ECNL)). Clubs within these platforms are not directly affiliated with any professional (i.e., NWSL) clubs, and players are free to play for multiple clubs across different platforms, which is likely in conjunction with their high school soccer team. Many clubs within these platforms typically operate a pay-to-play model, where club membership, registration fees, equipment, and travel can cost thousands of dollars per annum, potentially creating systematic barriers for players from less affluent households [37,38]. Furthermore, youth league platforms are not governed by the US Soccer Federation (USSF), they operate independently, setting their own rules, regulations, and philosophies [39]. Talent pathways in South America and Europe are relatively linear, players are eligible to join professional clubs’ youth teams around 10–11 years, where they transition through the age groups and potentially to the first/senior team [25]. During this time clubs provide their players with free practice and resources (e.g., equipment), and they are governed by their respective SFs [40], Following high school graduation, TD pathways differ further, with players often competing in domestic collegiate soccer leagues between 18–22 years under the auspices of the National Collegiate Athletic Association (NCAA). As student athletes, players compete in inter-collegiate soccer whilst enrolled on education courses at the university [37,38]. While this is aligned with North American SFs such as Canada [26], they differ from Europe where at similar ages players are playing at professional level [25]. Furthermore, youth-to-senior transitions occur within the same club (i.e., U18 to U23 to Senior/First teams). Yet until 2024, players in the US progressed to the professional game via a draft system, which involves NWSL teams selecting players from college soccer programs. However, recently there has been an increasing number of players opting to transition from the youth-to-professional level (e.g., Olivia Multrie), thus removing a previously significant component of their TD pathway.

Notwithstanding the popularity of soccer and success of the national teams, there is limited research examining environmental factors, and their impact on the TI and TD of youth female soccer players in the US [37,41–43]. Exploring the soccer environment and the unique constraints such as demographics (size; population), socio-economics (facilities; pay-to-play) and pathways (multiple league platforms; college soccer) would allow for a better understanding of the existing structure of youth soccer in the US at a national level. This will allow for more bespoke research that can provide stakeholders responsible for identifying potential youth national team (WYNT) players (e.g., scouts) with evidence informed information to support the talent process. Therefore, the aim of the present study was to examine the environmental landscape of youth female soccer in the US, and the impact of these constraints on TI and TD for WYNT players.

## Materials and methods

A convenience sample approach was adopted [44], with the potential sample known *a priori* due to their role (i.e., scout) within the US Soccer Federation (USSF). The USSF employ scouts across the US, who are responsible for TI within specific geographic areas and have experience in identifying female soccer players for the WYNT. The study was designed in accordance with the Declaration of Helsinki and approved by the lead author’s university ethics board (Table 1).

**Table 1:** Participant characteristics.

Characteristic		Mean (±)
*Age (years)*		44.5 (7.6)
*Scouting Experience (years)*		11.6 (8.8)
*USYNT Experience (years)*		9.7 (6.3)
		***n* (%)**
*Highest Coaching Qualification*	*USSF A Youth or Senior (Level 6/7)* *UEFA A (Level 4/5)* *USSF B (Level 5/7)* *USSF C (Level 4/7)*	17 (74) 4 (17) 1 (4) 1 (4)

***Note: USYNT = US Youth National Team; USSF = United States Soccer Federation***

### Procedure

All potential participants (*n* = 76) were identified by their job role and contacted via email by the lead author. The email contained a participant information sheet and consent form for consideration. After a two-week period, allowing for potential participants to decide whether they wished to be involved in the study, they were contacted again to determine their (non)willingness to participate [45]. A total of 23 scouts (13 female; 10 male) agreed to participate in the study and returned signed consent forms. Interviews with participants were arranged on dates and times suitable for participants. Due to the vast geographic distance of participants, it was determined that virtual interviews would be most appropriate [46], supporting efficient data collection processes and reducing burdens upon participants [47]. A semi-structured interview guide was developed in line with [48] (i.e., aligning interview questions with research questions; constructing an inquiry-based conversation based on previous literature [35–38]; receiving feedback on interview protocols from two co-authors (MJR; MvR); and piloting the interview protocol with one WYNT scout with 20 + years’ experience as a coach and scout, resulting in amendments to the wording and positioning of several questions to improve flow and reduce repetition and overlap). Interviews examined participants’ perceptions of the impact and influence of environmental factors upon TI and TD of female YNT players, broadly focused on. six overarching themes: (1) icebreaker questions that focused on the participants’ soccer background to promote trust and more authentic engagement from the participants during the interview process [45]; (2) demographics of US and (3) female youth soccer (i.e., birthplace effect; [49]); (4) the college system [38]; (5) multiple soccer platforms (e.g., ECNL/GA); and (6) the pay-to-play model in youth soccer [39]. These broad themes were underpinned by follow-up and probing questions to extract richer and more nuanced data by encouraging participants to expand on their thoughts and experiences [44,50].

### Semi-structured interviews

All interviews were conducted between November to December 2022, with an average length of 46.1 ± 8.3 mins. Prior to each interview starting, the purpose of the study and the interview procedure was clarified, participants were assured of anonymity, their right to withdraw at any time, confidentiality, and the intended use of the data collected. All interviews were conducted by the lead author. MvR provided an emic perspective to the data whilst MJR provided applied and theoretical considerations for the interview process. Interviews were conducted using Microsoft Teams (Microsoft, WA, USA), with audio recording and auto-transcribing enabled. Once an interview had been conducted data were checked for accuracy and consistency against the audio recording and cleaned (i.e., corrections to words, terminology, etc.). Once data had been checked and cleaned, they were included for analysis.

### Data analysis

Reflexive thematic analysis was adopted [51]. This approach allowed for inductive and deductive analysis to occur, enabling conceptualised patterns of shared meaning across the data [52]. Semantic coding generated (initial) themes, presenting the data as conversed by participants [53,54]. Codes were actively created [52], with the second author as an analytic resource, situated between the data and their contextual, theoretically embedded, interpretative practices. Repeated patterns were grouped in a meaningful way, with the refining, separation, and discarding of codes tracked using Microsoft Excel (Microsoft, WA, USA; [53,54]). This created a thematic map, providing insight into the significance of individual themes [55]. Coding quality came from the depth of engagement with the data and the situated, reflexive analysis process [56]. Information power was reached through a narrow study aim, a specific inclusion criteria for participants, the quality of dialogue in interviews, and the analysis conducted [57]. The relevance of themes was considered in relation to the research question and the broader data narrative, with theme titles generated and refined through discussion and critique by the authors during the write up of the study’s findings [52].

### Data integrity

This study sought to understand individual’s experiences of the identifying talent within the WYNT talent pathway. Employing a constructivist epistemology, we viewed knowledge as the product of social interactions and negotiations [56]. Ontologically, we took a relativist position, where the focus was on constructed, more than found, worlds [56]. Here, social reality was regarded as a product of how scouts, both individually and collectively, made sense of the varied factors and stakeholders within their social world [58,59]. Consequently, the interpretative, qualitative methods reported in this study offer a representation of reality by revealing an interconnected, multi-dimensional narrative experienced by participants (Salla, 1993) [60]. This work offers a substantive contribution to the literature, and we have strived to be transparent, providing a clear outline of our methods and analyses [61]. The knowledge and experience of the research team influenced interest in the research questions. The authors acted as critical friends to one another to support the application of methods, structuring the data collection/analysis, and providing researcher reflexivity [62].

## Findings and discussion

This study examined WYNT scouts’ perceptions of the impact of environmental factors upon talent TI and TD. Themes generated during the analysis were: (1) no established playing style; (2) finding players for WYNT; (3) players’ access to TI and TD opportunities; (4) limited involvement of the USSF in youth development.

### No established playing style and approaches to coaching

Notions of standardised playing style and approaches to coaching amongst clubs and WYNTs was highlighted as a missed opportunity for the USSF to be clear on “this is what we think is best” (P13). Across Europe, over the last decade, there have been concerted efforts by clubs and soccer federations (SF) to develop a playing style; typically, referred to as a philosophy, methodology, or DNA. For example, in December 2014, the England DNA was launched, that aimed at “creating winning England teams” (The Football Association, [63]). As part of the English FA’s approach, their framework included: (1) who they are; (2) how they play; (3) the future player; (4) how they coach; and (5) how they can support this process. A survey of male soccer clubs reported that 76% of clubs (out of 29) utilise an internal philosophy to support their TI and TD processes, and ~50% of clubs stated that between the ages of 8–16 years, a key objective was to create a ‘playing style’ [32]. Soccer can be identified by key ‘moments’ of the game that repeat during competition (e.g., defending transition; [64,65]), breaking these moments down can allow clubs or SFs to define their philosophy and/or playing style, thus providing key criteria that allows them to effectively identify, monitor and (de)select players [66]. It is notable that recently USSF introduced the ‘US Way’ playing philosophy, with the aim to place the player at the centre of every decision [67], thus it may be important for future research to examine its impact on TI and TD processes.

Participants highlighted that geographic differences influenced team playing styles, for instance where those in colder climates (e.g., Minnesota; Michigan) were better at playing in “tighter spaces”; influenced by the size/shape of training facilities available, and need to play indoors due to the weather, thus, how teams trained was “tied to field space” (P5). A previous study of male youth players from a small island SF found that training facilities shaped players’ development opportunities, leading to players engaging in higher levels of individual play over team-based coach-led practice [68]. Conversely, players from other areas were perceived to demonstrate ‘more flair to their game’, with ‘more athletic’ players (P2), compared to other ‘gritty’ (P4) areas. Indeed, participants described the US soccer environment as a ‘melting pot of different cultures’ with considerable influence from the Europe and South America. Therefore, club coaches held different ‘mentalities’ and ‘visions’ of how the game is played (P12), creating these regional differences in playing style across the country. Here, nuances between regions become more important ‘than differences between platforms’ (P6). As below:

I went and coached this summer in Spain […] and they all play the same. They all are looking at the game and looking at the exact same moments as a coaching staff [...] You can identify the best players on the field once they tell you, “This is how we train.” They’re like all on the same page […] they [players] hear the exact same thing until they play pro. The exact same methodology the whole way. [...] You go to Spain, you know what those players should look like. (P20)

TI is grounded in the subjective opinions of coaches [69]. Therefore, if like in Spain, coaches and scouts in the US had an agreed upon, aligned criteria (i.e., shared mental model) for the specific purpose of evaluating players, this would increase the quality of their identification and (de)selection processes [66,70]. This unified approach to player development was suggested to be further impacted by “only training one or two weeks together at a time” which “doesn’t allow cohesiveness with our YNTs and our WNT” (P2). Access to players is not a unique issue to the US, however, due to the country’s size and population it is considered inhibited from the creation of a “national identity” (P12), as many players are only able to meet regionally (25 events-per-year) and meet as a NT several times per year [43]. This created an apparent reliance on “really strong” individuals who “make up for a lot of difference […] without a true team style of play” (P9). Moreover, participants conceded that soccer was not “woven within the fabric of [US] culture” like it is in other countries but was something they were still “working on” (P22). Thus, the current ecosystem meant the proliferation of these varied and multiple playing styles.

The game model used on both coasts versus central are totally different, and so I think the struggle that our state has had is the tactical side when it comes to the game […] the west likes to play, they play more of a possession based versus ours is very direct and athletic in the south. There’s a mishmash on the east coast […] technically it’s clean, but they don’t have the same athletes that we have in the south. (P20).

### Finding players for Women’s Youth National Team

Participants discussed ‘continually looking’ for players including those ‘kids who aren’t noticed’ (P7) to build player numbers for the NT. Players at the youth level travelled a lot, with some families driving ‘two hours, four times-a-week’ for their child to practice (P20). Players travel ‘four- and six-hours’ journeys (P6) – others driving ‘6 or 8 or 10 hours’ (P7) – to engage with soccer activities; and with a sparse mass transit system in the US, most rely on personal transport or flights. A study of male players reported low-moderate cognitive fatigue being associated with long travel to training and competition [71]. The issue of travel is reflective of broader systematic issues associated with the structure of soccer in the US compared to an ‘ideal type’ model that also includes accessibility to high-quality coaching and facilities, as well as socio-economic challenges [35,37].

Participants discussed how they frequently saw players “play in more than one state” per month (P13); and how their performances often seemed to be inconsistent. Participants detailed how they had seen and heard of numerous players becoming injured due to extensive travel where players “get off the plane and run to the field” (P1). However, some participants also suggested that such travel was necessary for player development, enabling competition against different teams than the limited number with whom they would more often compete. Indeed, previous studies have found that other situational variables are more influential upon performance than short-haul air travel such as match location (i.e., home teams in sports competition win over 50% of games), tactics (i.e., formations, playing positions), and players psychological state (e.g., players perceptions of tiredness, sleep quality, irritability) [72–75].

If you changed it and you went back to just playing teams in your own state, what’s gonna happen? The development’s gonna falter because you’re not gonna get to play against teams and players that are at the same level […]. It is what it is you pay to put your child into a more competitive environment, and if that’s not what you wanna do, you get the choice to not do it. (P23)

Studies have suggested that engagement in challenging competition may be an important developmental activity for future success in sport [23,25,26]. Thus, those who can afford such opportunities, gain access to better ‘player education’, ‘player practice’, in environments that attract “better players” (P15). However, the influence of soccer and, thus, the diversity of players was suggested to vary across states, highlighting how culture and players’ upbringing impacted why players were identified. Youth academy male soccer players in England perceived as high-potential came from lower socio-economic family backgrounds compared to their low-potential peers [20]. However, an examination of birthplace in professional female soccer players in Brazil between 2003–2020 indicated an overrepresentation (~80%) of players born in states with the highest Human Development Index tertile [19]. It is imperative to acknowledge the specific socio-cultural norms differ between countries such as population size and density, participation rates, strength of domestic competition, access to sports facilities, better coaching guidance, and financial and logistical resources [29]. Furthermore, the contribution of socio-economic factors in TD are often overlooked [8]. For example, an analysis of the geographical distribution of national team soccer players in Italy indicated an overrepresentation of players from the north Italy, due to disparities that affect Italy from a socio-cultural and economic point of view [76]. Like the US, youth soccer in Italy operates on a pay-to-play model (i.e., fees that cover membership/registration fees, equipment, and travel [37,39]). A ‘talent map’ of youth female soccer in the US may offer a greater understanding of the socio-cultural factors such as the birthplace effect [77].

The ‘lack of opportunities’ for players to play against other ‘highly skilled’ as players as part of the NT was suggested to inhibit the identification of players (P23). When in a US Soccer environment (e.g., TI center), it offered scouts better opportunities to ‘bring the highest talented players’ and evaluate them (P11) compared to their club settings, providing a more challenging environment to identify and evaluate their ‘key qualities in one singular environment’ (P9) because talent looks different in different spaces. Participants always considered how ‘good’ players will look ‘around better players?’(P4).

We sometimes find what I was saying looks like a talented player who may indeed dominate play but who has relied on their strength, their speed, their athleticism […]. They’re still good tactically. And technically, but are they superior? […]. Now when that same player is put in an environment and an IDC or YNT, suddenly they don’t look as dominant. (P6)

TI centers, though, are restricted by the cost of human and physical resources. The multiple ‘platforms’ that players compete in weekly often results in scouts ‘missing players’ as ‘it’s impossible’ to ‘see everyone’ (P11). Participants expanded how the process ‘should start’ from an ‘unbiased’ position as US Soccer was objective with clubs when they recommend players to attend a TI event. The use of subjective opinions of ‘experts’ have previously been employed [78]. But these ratings can be (un)consciously biased towards older, more mature players [79,80]. Indeed, 29% of youth female ‘club level’ players observed by US WYNT scouts were early-matures (biological age < chronological age; [43]). However, many player observations are a ‘snapshot’ (P22) in isolation; as participants explained, scouts ‘keep coming back to’ their ‘evaluation of [the player’s] performance on the day’ (P23), sometimes basing ‘decisions on 45 minutes’ of play (P10). This negative perception of singular observations implied by the participants is consistent with scientific literature [66], and thus it could be suggested that scouts assess/evaluate across multiple timepoints that has been advocated [3,81], as it can serve as a monitoring tool for a group of players. Single, rushed observation/identification can lead to players being ‘pigeonholed’ into positions ‘they’re not naturally comfortable’ to ‘satisfy’ the ‘will of the coach’ (P12). This is outlined in the below quote:

I was out and it was a player I had seen at an IDC, and when we saw her in the IDC, most of us there felt like she was a 10, maybe an 11. But the coach plays six. […] He was like, “look within my team, if I don’t play as the six, the opponent is gonna run through my midfield. I need her there” [...]. Understanding that context, I didn’t walk into the game, I think otherwise I may have gone. “Why does she not dribble forward? why does she not penetrate?” [...] Because I would say her standout qualities were a lot of attacking qualities […]. Understanding her decision-making helped me not describe her as hesitant. (P6)

The contextualisation of talent is further complicated by the future-focused aspect of the scout’s role. They are sometimes ‘projecting’ and finding the best player for the future, rather than focusing on ‘standout players’ (P15) who are the best current player [32]. Outlined below:

I kept saying, you know, “She’s not good enough” and somebody said “yet”. That simple word of ‘yet’ flipped my entire mindset of what we’re trying to do as YNT scouts. […] We make a lot of assumptions based on player actions of young players. […] We were watching a game this weekend and somebody’s like, she’s […] not good enough at solving a 3-back system, and my thought was “how in the world do you know that? […] in this limited snapshot of a game?” (P14)

Large numbers of players in the system leaves a large ‘scope of development’ because some players ‘might reach their potential later’ (P7) with ‘everybody [getting] there at their own pace’ (P1). This makes it difficult for the WYNT scouts as they are indirectly predicating a player’s potential future performance whilst presuming that the best ‘current’ performers are also the ones with the highest potential [82]. However, while the role of a WYNT scout is to recognise and select players with the highest potential to contribute the associations future success [83] at senior levels (i.e., WNT), they are also under pressure to succeed at youth levels (e.g., U17 FIFA World Cup). While finding that ‘diamond in the rough is very, very difficult’ (P15), the ‘demarcation’ between players ‘is so small’ (P5). An example of this can be seen in the below quote:

As you are trying to assess a player’s […] rating, they’re making decisions based on their position, which we have our positional traits and our key qualities, and if we’re not seeing them make those decisions then it becomes subjective. Are they doing it because they don’t know what to do or they not doing it or doing it because that’s their style of play? […] If that style of play for that team is a low block, we’re just gonna drop off and let that team come and just shift side to side, then you’re not going to see explosive defending pressure from that player. [...] Now I’m not seeing that talent of explosive physical execution. So now I’m like, “OK, does a player have it or not have it?” [...] You’re trying to understand all these facts to create a rating based on that game. (P15)

US Soccer have processes, which fitted talent into the system. There was an established ‘common language’ and ‘templates’ to identify the ‘benchmarks’ scouts should ‘look for’ (P13), providing ‘some direction’ (P17):

You start articulating things better, you start to see things better. The overall opinion and the overall assessment of that player is, yeah, she’s good. […] so, now you have your key points to why you’re getting there. […] The key characteristics have been important in that. (P17)

Observed players current and potential skills are rated by scouts using a ‘scout submission’ containing key skills allied with position-specific key qualities [43]. This approach of scouts scoring specific soccer skills rather than an overall impression has been previously proposed to increase the quality of the (de)selection process of a player [70], as it allows for a more structured data collection than a traditional unstructured holistic approach [71,82]. Although ‘helpful’ in outlining ‘what US Soccer wanted’ (P23) in terms of a benchmark, every scout may have a ‘different lens’ of what they see as ‘talent’ [83,84], leaving the guidance ‘a foundation’ of scouts ‘subjective biases’ (P15) that may be underpinned by their intuition and/or practical experience [69,85] that impacts scouts’ ‘idea of what talent is’ (P20), as highlighted below:

A player can be talented […] but if they don’t fit the needs of a 10 within the system that the US is playing, then they may not rise to the top […] What does US Soccer look for in a 10? In a 6? […] I start, kind of, going down what they are, and I try to make notes […] where are they positioning themselves to receive the ball? […] How are they verbally communicating with their teammates? Technically, how are they executing, especially under pressure? […] are they anticipating what’s going to happen? (P6)

One potential approach to address this issue is the introduction of a shared mental model [86]. In dynamic team environments, these models help align understanding and expectations among coaches, scouts, and support staff [87]. Previously, a shared mental model for TI was created in a professional boy’s academy in England [86]. Findings highlighted that coaches could use the themes to structure TI assessments, such that they are criteria based overly purely subjective, thus reducing potential biases, as well as allowing for longitudinal tracking of player development [86]. While this was based in a single academy, the methodologies employed are a model that can be potentially be replicated for youth female soccer environments in the US, enhancing their TI and TD processes through evidence-based, contextually grounded practices. Such research highlights one of the associated challenges of player evaluation [66]. It’s difficult to ‘score’ these players because ‘they don’t have exactly the qualities that we have listed and looking for’, even though they have ‘some special quality’ (P10). Yet it is important to ‘have variety’ and ‘not trying to find the same in every player’, fitting a structure, but also considering what will help US Soccer be successful in the future (P11). This highlights the ‘subjective’ nature of scouting, and the difficulties of potentially merging with ‘objective’ process (P13) such as motor tests, that have been shown to be utilised in high performing youth female soccer clubs in Europe (e.g., [88]) and offer the best predictive value of future performance [89]. Whilst the importance of the role of scouts play in the TI process is well known [32], there is a need to further explore the criteria of which these ‘scores’ are based [21,82,90,91], particularly in youth soccer in the US. The process also risked ‘all this data’ and ‘very few players that really meet’ the key qualities (P8) and limited opportunity to provide feedback to players because of ‘the sheer number of people’ to provide feedback to (P10).

### Player’s access to talent opportunities

TD environments are one of the most directly controllable factors associated with determining a player’s development and future success [92]. Consistent with the literature surrounding the birthplace effect in soccer [76,77,93], scouts discussed how more densely populated areas produce the right environments for players and how this differed across regions. Linking to the preceding two themes, areas of the US with more developed cultures of soccer tend to ‘breed more professional clubs’ and ‘competitiveness’(P23). This leaves players in smaller states having to work ‘harder to get into the same environment’ because a bias has already ‘been established’ (P23). Being in one of these ‘lesser populated’ states ‘stunts’ player development because a lack of ‘competitive environment’ inhibits players’ abilities to ‘grow into what US Soccer is looking for’ (P23). This perception of scouts favouring more densely populated areas is varies from previous reports of birthplace (e.g., [94]), suggesting that they provide less facility access and support [18]. Teoldo et al. [19] reported professional female soccer players in Brazil were more likely to be born in either small less populated areas (20%), or large densely populated (15%) areas. As the birthplace effects vary between nations and sports [77], this further warrants the need to explore a ‘talent map’ of youth female soccer in the US [43]. Moreover, scouts continue to highlight that ‘natural’ talent is not enough, highlighting the importance of ‘access to good competition on a week-to-week basis’s (P12). Sometimes, accessing the right environment means being identified as talented. Participants discussed the pressure on early identification because the ‘top 5-10%’ (P10) of players identified early will receive financial aid packages from clubs, as shown from the below quote:

If you’re a very, very good player, you’re probably not paying […] clubs really want you to play. I don’t think you’re paying to play there. […] Those YNT players know they’re gonna get a full scholarship to places that they will want to go […] I think that if you’re good at soccer, someone will find you and give you a scholarship or waive the fees and you’ll be able to play at a top club (P10).

This meant parents can feel (an unnecessary) pressure to have players seen. Early identification brings challenges for players: ‘Suddenly they’re looked upon’ differently and parents start making ‘adjustments’ (P6) because ‘someone who is an expert […] told them that their kid has something’, which can lead to ‘attrition’, isolating players from the ‘other kids who may be on [the same] path’ (P6). Conversely, not being identified can mean players ‘lack motivation to keep going on their journey’ feeling they have ‘no chance’, perhaps exploring ‘other sports’ or engage with soccer ‘more recreationally’ (P11). Although specialising early (engaging in high intensity practice within a single sport) is correlated with negative long-term outcomes (injury, overtraining, burnout; [95]), the pressure on youth players (and parents) to specialise as early as possible to accumulate high amounts of soccer-specific practice, and potentially increase their chances of being identified is difficult to avoid [96]. It could be suggested that like professional players in England, they engage in higher amounts of peer-led-play during childhood [25].

Furthermore, scouts highlighted the issues surrounding the cost of engaging in youth soccer in the US and the links to TI. The dilemma: ‘You have to have money to even get identified’ (P14). Some families are paying ‘three to four thousand dollars per year in club and team fees’ (P11), which could ‘easily’ increase to ‘ten to fifteen’ a year’ (P17) and sometimes ‘close to twenty-five’ (P1) or ‘thirty a year’ (P15). This creates an inaccessible system where ‘fantastically talented kids’ who ‘just can’t afford it’ are missed (P1). Previous studies have shown a relationship between country socio-economics and athlete development [11,76,97,98]. For instance, professional players in the NWSL spent their formative years in suburban, socio-economically advantaged (i.e., high per capita, median household, and family incomes) areas than the national average [37]. Moreover, ‘a lot of families will self-select’ out of soccer ‘before they even have a chance to find out about those scholarship situations’ based on ‘how expensive it is to play’ (P12). Consistent with global disparities in financial investment between men’s and women’s soccer [99], most financial support is ‘on the boy’s side’ of the game, with little offers for female players (P15).

Every club will say, “well, we’re not preventing anybody from being a part of this because we have financial aid”, but there are some big obstacles to securing financial aid. There’s a lot of parents who don’t/wouldn’t qualify for financial aid, but still can’t afford those kinds of cost [...] When they’re deciding on a club […] they’re going to play for where their parents can afford. (P9)

For those who can afford to be part of a club, participants identified numerous factors that impact players hours in practice. First, differences in weather climates across the US presents a bigger challenge than ‘we assume’ with the impact on training frequency in appropriate spaces ‘understated’ and ‘underexamined’ (P8). In some regions, players are not outside ‘11 months a year’ and when they are, the expense means ‘small-sided games’ and ‘playing’ with ‘not a lot of teaching’ is seen as the most cost-effective use of the space (P1). Some participants based in regions with colder winters shared how indoor facilities help clubs train year-round, competing with warmer climates; however, this was not an option for everyone. These differences in opportunities to practice due to weather/facilities may lead to interindividual variation in soccer-specific engagement, consistent with reports in female soccer players from high-preforming nations including the US [25,27]. Moreover, players that practice indoors typically do so on small pitches, which although develops facilities their technical execution, it may limit their physical skill development, which is then ‘tested’ when playing in ‘expansive spaces’ (P22). Further, development was discussed as ‘really’ coming from ‘playing games’, ‘trying things’ and ‘learning from it’ (P10).

[These] kids aren’t learning how to defend, they’re not learning how to shoot, they’re not learning how to do the developmental piece. We don’t practice on a big field because there’s four teams on [the] field. There’s no way you can […] understand the game when you’re playing on a 60 by 40. (P7)

The final aspect of players’ opportunities linked to the collegiate system. Participants discussed how this is envied by ‘people around the world’ (P4), where some college facilities are ‘better than professional clubs’ (P17) providing ‘300 and something pro environments just in Division One College soccer’ (P2). Furthermore, these colleges provide desirable financial support via scholarships as well as significant investment in athlete support and is a major pull for players inside and outside of the US [37,38,40,100,101]. Despite this, the college system has limitations such as a truncated playing and training schedule (17 games, 25 practices in 56 days), differing rules (e.g., substitutions, overtime etc.), and limited roster numbers [102]. Complicating the scout role, some colleges are recruiting players aged ~15, which pushes players at a ‘younger age to perform at a high level’ (P20), further promoting early specialisation [95]. In addition, participants shared how players are in a competitive, ‘professional environment’ between 18–22 years old (P23). This can provide invaluable time for physical and emotional development, with peers of a similar age, before exiting and joining a professional team of older adults. In addition, it is a system that promotes personal development:

Those young women also know the value of an education and that they, when they’re playing careers over, they want to have something to fall back on and be able to go out and get a great job […] it’s all under one umbrella […] the professors and the coaches work together, you’ve got sports medicine support like psychology, strength the conditioning, nutrition, you’ve got all the resources you would have as a professional [...] You’re allowed to pursue your sporting dreams and desires while you’re getting a great education at the same time. (P12)

### Limited involvement of US Soccer in youth development

In relation to TD, participants discussed attempts to create ‘equal’ systems ‘from top down’ (P14) across the male and female game, but recognised the female game had a different infrastructure. Participants lacked a focus on developing talent, leaving TI centers a place to ‘eyeball’ players, ‘put them through a training session’ playing ‘11 vs. 11’, and then ‘send them on their way’ (P1). Consequently, scouts were ‘not in the trenches developing’ players, meaning ‘the quality of players’ is ‘completely on the clubs’ (P1). In addition, participants discussed how clubs become powerless to the culturally accepted pressure to win, with ‘everyone wanting to be better than everyone else’ (P11). This ‘competitive mentality’ means ‘wanting to compete and win’ becomes ‘a very important skill’, woven throughout the US culture, which can be ‘positive for talent ID’ (P11). However, it can focus on short-term development, rather than the longer term.

They’re not really looking at it (player development) long term. They’re in a rush. […] connecting back to the pay to play, they’ve gotta make their revenue with their players when they can in there because again, the clock stops for most clubs at 18. (P8)

The prospects of winning ‘draws’ parents in (P14), yet it can impact TD because players ‘have to perform’ or their ‘season’s going to be cut short’ (P10) [35]. Clubs focused on winning lose focus on player learning (i.e., TD), searching for players to ‘win games right away’ (P14). These perceptions may suggest that coaches within these platforms feel pressure to succeed in the short-term and thus may select older players [42,43] who may the best current performers, rather than the most promising players long-term [9]. However, this culture also leads to clubs bowing to ‘pressure from parents’: ‘If you don’t do what your best player feels like you need to do, or you’re not winning the games, your best player may jump to your nearest opponent right next door’ (P10). Therefore, ‘good parents’ with ‘a lot of money, influence the clubs’ (P7), creating competition between clubs.

This means scouts become disconnected from players, assuming all players’ goals are to ‘make it to the YNTs’, ‘play professionally’, and make the WNT, which might be ‘false’ (P8). The women’s ‘culture’ in the US is ‘reflected by what the college coaches want’ (P1). Consequently, most clubs ‘are geared towards the college game’ (P1), ‘tied into the fact [players] want a college scholarship’ (P17). It’s the ‘primary, number one’ focus (P1), which ‘sells the club’ (P2), but means clubs are not ‘held accountable for the quality of the player’ (P1). For some, college soccer becomes a steppingstone, with participants discussing how players focus on the collegiate system and then ‘along the way’ discover they are ‘good enough’ to be a national team player (P7). Crucially, however, participants discussed that the college ‘style of play is not conducive to making national team players’ (P1). Moreover, some participants were concerned players only realistically received ‘132 days’ of contact time with coaches, meaning they are responsible for their own training for ‘two thirds’ of the year, which ‘maybe 1% of those kids are doing’ (P22).

There are multiple platforms that US soccer does not influence, but participants discussed how channelling players into two platforms is ‘affecting youth player development’ (P9). The scale of the US impacts the ability to bring ‘everyone on the same page’ (P11). Scouts raised concerns associated with coaching qualification and there was still ‘a lot of average coaches’ developing ‘future WYNT players’ (P14), with the ‘best coaches’ coaching the senior teams rather than grassroots because some coaches perceived it as les of a challenge to coach girls rather than boys’, meaning ‘the best coaches’ were not ‘coaching female soccer players’ (P14). The result is’12–20 clubs’ that ‘produce high calibre players’ (P10). Yet participants discussed ‘more talent’ as the result of ‘better coaches over the last 30 years’ delivering a ‘higher percentage of quality coaching’ (P13). This means players receive ‘better instruction at younger ages’ (P23).

There’s a lot of former players that are now coaching. […] Now our players have the benefit of having the experienced player [or] coach and the ability to attend courses and the ability to read literature regarding […] different types of coaching trends and then also the international game I think has come into our systems in some way, shape and form […] it turns it into a bit of a melting pot. (P23)

Part of this improved coaching was attributed to the “pay-to-play” system, which allows coaches to ‘make a living as a professional coach’ in the youth game (P14). Yet some participants were concerned that ‘poser coaches’, who are ‘there to get paid’, could hinder player development (P7).

## Conclusion

This study provides valuable insight into the key environmental factors influencing TI and TD in youth female soccer in the US, through the lens of WYNT scouts. Major themes included the inconsistency in playing styles across regions, impact of demographic diversity and logistical challenges (e.g., travel, time with WYNT), disparities in access to TI and TD opportunities, and the limited involvement of the USSF. These environmental dynamics represent critical factors shaping identification and progression of youth female players within one of the world’s most successful yet under-researched soccer systems.

By focusing specifically on the US female context, this study addresses a significant gap in the applied literature and offers actionable recommendations to improve TI and TD processes nationwide. For instance, we advocate for the co-development of a shared mental model across stakeholders to create an evidence-informed framework that defines key position-specific skills and performance indicators that ensures alignment in playing philosophy and reliability in TI and selection processes [103]. Future steps could include using the themes within the present study as a foundation for the framework, working with key stakeholders (e.g., coaches, scouts, sport scientists) to develop agreed performance indicators and terminology (e.g., anticipation), as well as methods to collect data that reflects the shared mental model [86]. Moreover, we propose the creation of a national ‘talent map’, incorporating environmental variables such as birthplace, socio-economic background, education, and family structure [8] to help identify potential barriers to participation and inform strategic planning. To enhance objectivity in player evaluation, we emphasise the need for multi-game, longitudinal assessments supported by data. While this approach has demonstrated predictive validity in male youth soccer (e.g., [104,105]), it remains unexplored in the female game, a gap that warrants urgent attention. In addition, expanding and equalising access to scouting nationwide is essential to ensuring a broader and more inclusive player pool. Beyond these recommendations, our findings suggest further investment in coach education, particularly at the grassroots level, to equip coaches with tools to identify and nurture emerging talent. For example, previous studies have shown that grassroot and high-level coaches prefer gaining craft knowledge from informal sources such as social media and the internet [103,106]. Therefore, similar tools could be adopted to equip grassroots coaches with the knowledge regarding relative age effect that is current missing in the US and Germany [43,107]. Together, these initiatives could drive more equitable and effective development pathways and strengthen the talent pipeline in women’s soccer.

It is important to note that while this study draws from the experiences of 23 scouts (~25% of the WYNT scouting base at the time of data collection), the findings represent the perspectives of a specific cohort and may not capture the full diversity of regional and club-based practices. Nonetheless, the contextual richness of this data contributes to a deeper understanding of the unique challenges and opportunities within US youth female soccer. As one of the first studies to focus on this demographic in a high-performing yet under-researched federation [108], it provides an important foundation for future inquiry and evidence-informed practice.

## References

[1] FIFA. Women’s Football: Member Associations Survey Report 2023. 2023. Available from: https://digitalhub.fifa.com/m/28ed34bd888832a8/original/FIFA-Women-s-Football-MA-Survey-Report-2023.pdf

[2] FIFA. Women’s Football Strategy. Available from: https://digitalhub.fifa.com/m/baafcb84f1b54a8/original/z7w21ghir8jb9tguvbcq-pdf.pdf. 2018. Accessed 2025 February 20.

[3] Williams AM, Ford PR, Drust B. Talent identification and development in soccer since the millennium. J Sports Sci. 2020;38(11–12):1199–210. doi: 10.1080/02640414.2020.1766647 32568000

[4] Okholm Kryger K, Wang A, Mehta R, Impellizzeri FM, Massey A, McCall A. Research on women’s football: a scoping review. Sci Med Footb. 2022;6(5):549–58. doi: 10.1080/24733938.2020.1868560 36540910

[5] Emmonds S, Heyward O, Jones B. The Challenge of Applying and Undertaking Research in Female Sport. Sports Med Open. 2019;5(1):51. doi: 10.1186/s40798-019-0224-x 31832880 PMC6908527

[6] Curran O, MacNamara A, Passmore D. What About the Girls? Exploring the Gender Data Gap in Talent Development. Front Sports Act Living. 2019;1:3. doi: 10.3389/fspor.2019.00003 33344927 PMC7739739

[7] Peters CM, Hendry DT, Hodges NJ. A scoping review on developmental activities of girls’ and women’s sports. Front Sports Act Living. 2022;4:903886. doi: 10.3389/fspor.2022.903886 36213454 PMC9538116

[8] Rees T, Hardy L, Güllich A, Abernethy B, Côté J, Woodman T, et al. The Great British Medalists Project: A Review of Current Knowledge on the Development of the World’s Best Sporting Talent. Sports Med. 2016;46(8):1041–58. doi: 10.1007/s40279-016-0476-2 26842017 PMC4963454

[9] Sarmento H, Anguera MT, Pereira A, Araújo D. Talent Identification and Development in Male Football: A Systematic Review. Sports Med. 2018;48(4):907–31. doi: 10.1007/s40279-017-0851-7 29299878

[10] Roberts SJ, McRobert AP, Rudd J, Enright K, Reeves MJ. Research in Another un-Examined (RAE) context. A chronology of 35 years of relative age effect research in soccer: is it time to move on? Sci Med Footb. 2021;5(4):301–9. doi: 10.1080/24733938.2020.1841278 35077305

[11] Andrew M, Finnegan L, Datson N, Dugdale JH. Men Are from Quartile One, Women Are from? Relative Age Effect in European Soccer and the Influence of Age, Success, and Playing Status. Children (Basel). 2022;9(11):1747. doi: 10.3390/children9111747 36421196 PMC9689054

[12] McAuley ABT, Hughes DC, Tsaprouni LG, Varley I, Suraci B, Roos TR, et al. Genetic association research in football: A systematic review. Eur J Sport Sci. 2021;21(5):714–52. doi: 10.1080/17461391.2020.1776401 32466725

[13] Varillas-Delgado D, Del Coso J, Gutiérrez-Hellín J, Aguilar-Navarro M, Muñoz A, Maestro A, et al. Genetics and sports performance: the present and future in the identification of talent for sports based on DNA testing. Eur J Appl Physiol. 2022;122(8):1811–30. doi: 10.1007/s00421-022-04945-z 35428907 PMC9012664

[14] Kelly AL, Williams CA. Physical Characteristics and the Talent Identification and Development Processes in Male Youth Soccer: A Narrative Review. Strength & Conditioning Journal. 2020;42(6):15–34. doi: 10.1519/ssc.0000000000000576

[15] Dugdale JH, Myers T, Sanders D, Andrew M, Clarke R, Hunter AM. Evaluation of multi-directional speed qualities throughout adolescence in youth soccer: The non-linear nature of transfer. J Sports Sci. 2024;42(4):301–12. doi: 10.1080/02640414.2024.2329846 38484363

[16] MacNamara Á, Button A, Collins D. The Role of Psychological Characteristics in Facilitating the Pathway to Elite Performance Part 1: Identifying Mental Skills and Behaviors. The Sport Psychologist. 2010;24(1):52–73. doi: 10.1123/tsp.24.1.52

[17] MacNamara Á, Button A, Collins D. The Role of Psychological Characteristics in Facilitating the Pathway to Elite Performance Part 2: Examining Environmental and Stage-Related Differences in Skills and Behaviors. The Sport Psychologist. 2010;24(1):74–96. doi: 10.1123/tsp.24.1.74

[18] Côté J, Macdonald DJ, Baker J, Abernethy B. When “where” is more important than “when”: birthplace and birthdate effects on the achievement of sporting expertise. J Sports Sci. 2006;24(10):1065–73. doi: 10.1080/02640410500432490 17115521

[19] Teoldo I, Machado VR, Casanova F, Cardoso F. Talent map of female soccer: How does the birthplace and birthdate impact the participation of soccer players in Brazilian Serie A1 Championship? JHSE. 2023;18(4). doi: 10.14198/jhse.2023.184.10

[20] Kelly AL, Williams CA, Jackson DT, Turnnidge J, Reeves MJ, Dugdale JH, et al. Exploring the role of socioeconomic status and psychological characteristics on talent development in an English soccer academy. Sci Med Footb. 2024;8(3):251–9. doi: 10.1080/24733938.2023.2213191 37161818

[21] Reeves MJ, Roberts SJ, McRobert AP, Littlewood MA. Factors affecting the identification of talented junior-elite footballers: a case study. Soccer & Society. 2018;1–16. doi: 10.1080/14660970.2018.1432383

[22] Côté J. The Influence of the Family in the Development of Talent in Sport. The Sport Psychologist. 1999;13(4):395–417. doi: 10.1123/tsp.13.4.395

[23] Gledhill A, Harwood C. Developmental experiences of elite female youth soccer players. International Journal of Sport and Exercise Psychology. 2014;12(2):150–65. doi: 10.1080/1612197x.2014.880259

[24] Güllich A. “Macro-structure” of developmental participation histories and “micro-structure” of practice of German female world-class and national-class football players. J Sports Sci. 2019;37(12):1347–55. doi: 10.1080/02640414.2018.1558744 30582400

[25] Andrew M, Ford PR, Alder SE, Champ FM, Brownlee TE, Datson N, et al. Talent development in female soccer: Developmental activities of professional players in England. J Sports Sci. 2024;1–12. doi: 10.1080/02640414.2024.2356434 38916272

[26] Hendry DT, Williams AM, Ford PR, Hodges NJ. Developmental activities and perceptions of challenge for National and Varsity women soccer players in Canada. Psychology of Sport and Exercise. 2019;43:210–8. doi: 10.1016/j.psychsport.2019.02.008

[27] Ford PR, Hodges NJ, Broadbent D, O’Connor D, Scott D, Datson N, et al. The developmental and professional activities of female international soccer players from five high-performing nations. J Sports Sci. 2020;38(11–12):1432–40. doi: 10.1080/02640414.2020.1789384 32627682

[28] Baker J, Cobley S, Schorer J. Talent Identification and Development in Sport: International Perspectives. International Journal of Sports Science & Coaching. 2012;7(1):177–80. doi: 10.1260/1747-9541.7.1.177

[29] Bennett KJM, Vaeyens R, Fransen J. Creating a framework for talent identification and development in emerging football nations. Science and Medicine in Football. 2018;3(1):36–42. doi: 10.1080/24733938.2018.1489141

[30] Baker J, Schorer J, Cobley S, Bräutigam H, Büsch D. Gender, depth of competition and relative age effects in team sports. Asian Journal of Exercise & Sports Science. 2009;6(1). 10.1111/j.1600-0838.2008.00838.x 18627551

[31] Romann M, Fuchslocher J. Influences of player nationality, playing position, and height on relative age effects at women’s under-17 FIFA World Cup. J Sports Sci. 2013;31(1):32–40. doi: 10.1080/02640414.2012.718442 22909307

[32] Ford PR, Bordonau JLD, Bonanno D, Tavares J, Groenendijk C, Fink C, et al. A survey of talent identification and development processes in the youth academies of professional soccer clubs from around the world. J Sports Sci. 2020;38(11–12):1269–78. doi: 10.1080/02640414.2020.1752440 32378447

[33] Simpson D, Martindale RL, Travlos A, Souglis A, Andronikos G. An investigation of the talent development pathway in Scottish female football. International Journal of Sport Psychology. 2022;53(3):218–41. doi: 10.7352/IJSP.2022.53.218

[34] Kelly AL, Eveleigh C, Bergmann F, Höner O, Braybrook K, Vahia D, et al. International perspectives: Evaluating male talent pathways from across the globe. Talent identification and development in youth soccer. Routledge. 2023:228–62.

[35] Smolianov P, Murphy J, McMahon SG, Naylor AH. Comparing the practices of US Soccer against a global model for integrated development of mass and high-performance sport. Managing Leisure. 2014;1–21. doi: 10.1080/13606719.2014.929402

[36] Stephenson EF. International Competitions, Star Players, and NWSL Attendance. Journal of Sports Economics. 2023;25(2):186–99. doi: 10.1177/15270025231217973

[37] Allison R, Barranco R. ‘A rich white kid sport?’ Hometown socioeconomic, racial, and geographic composition among U.S. women’s professional soccer players. Soccer & Society. 2020;22(5):457–69. doi: 10.1080/14660970.2020.1827231

[38] Markovits AS, Hellerman SL. Women’s soccer in the United States: Yet another American ‘Exceptionalism’. Soccer & Society. 2003;4(2–3):14–29. doi: 10.1080/14660970512331390805

[39] Pius Chukwunwike N, Mahmud O. Eradicating the pay-to-play system in American youth soccer: Economic, social, and athletic implications for grassroots development. World J Adv Res Rev. 2025;25(3):068–85. doi: 10.30574/wjarr.2025.25.3.0588

[40] Alder S, Causer J, Champ F, McRobert A, Datson N, Andrew M. Talent identification and development processes of female soccer academies from the top three tiers in England. Journal of Expertise. 2024;7(4):130–48.

[41] MacDonald DJ, King J, Côté J, Abernethy B. Birthplace effects on the development of female athletic talent. J Sci Med Sport. 2009;12(1):234–7. doi: 10.1016/j.jsams.2007.05.015 17889609

[42] Korgaokar AD, Farley RS, Fuller DK, Caputo JL. Relative Age Effect Among Elite Youth Female Soccer Players across the United States. SMJ. 2018;16(3):37–41. doi: 10.26773/smj.181007

[43] Finnegan L, van Rijbroek M, Oliva-Lozano JM, Cost R, Andrew M. Relative age effect across the talent identification process of youth female soccer players in the United States: Influence of birth year, position, biological maturation, and skill level. Biol Sport. 2024;41(4):241–51. doi: 10.5114/biolsport.2024.136085 39416506 PMC11475004

[44] Gray DE. Doing research in the real world.

[45] Bolderston A. Conducting a Research Interview. J Med Imaging Radiat Sci. 2012;43(1):66–76. doi: 10.1016/j.jmir.2011.12.002 31052024

[46] Lo Iacono V, Symonds P, Brown DHK. Skype as a Tool for Qualitative Research Interviews. Sociological Research Online. 2016;21(2):103–17. doi: 10.5153/sro.3952

[47] Braun V. Collecting qualitative data: A practical guide to textual, media and virtual techniques. Cambridge University Press. 2016.

[48] Castillo-Montoya M. Preparing for interview research: The interview protocol refinement framework. Qualitative Report. 2016;21(5).

[49] Morse JM. Critical Analysis of Strategies for Determining Rigor in Qualitative Inquiry. Qual Health Res. 2015;25(9):1212–22. doi: 10.1177/1049732315588501 26184336

[50] Robinson OC. Probing in qualitative research interviews: Theory and practice. Qualitative Research in Psychology. 2023;20(3):382–97. doi: 10.1080/14780887.2023.2238625

[51] Braun V, Clarke V. Reflecting on reflexive thematic analysis. Qualitative Research in Sport, Exercise and Health. 2019;11(4):589–97. doi: 10.1080/2159676x.2019.1628806

[52] Braun V, Clarke V. To saturate or not to saturate? Questioning data saturation as a useful concept for thematic analysis and sample-size rationales. Qualitative Research in Sport, Exercise and Health. 2019;13(2):201–16. doi: 10.1080/2159676x.2019.1704846

[53] Byrne D. A worked example of Braun and Clarke’s approach to reflexive thematic analysis. Qual Quant. 2021;56(3):1391–412. doi: 10.1007/s11135-021-01182-y

[54] Trainor LR, Bundon A. Developing the craft: reflexive accounts of doing reflexive thematic analysis. Qualitative Research in Sport, Exercise and Health. 2020;13(5):705–26. doi: 10.1080/2159676x.2020.1840423

[55] Braun V, Clarke V, Weate P. Using thematic analysis in sport and exercise research. Routledge handbook of qualitative research in sport and exercise. Routledge. 2016:213–27.

[56] Lather P. Critical inquiry in qualitative research: Feminist and poststructural perspectives: Science “after truth”. Foundations for research. Routledge. 2003:219–32.

[57] Malterud K, Siersma VD, Guassora AD. Sample Size in Qualitative Interview Studies: Guided by Information Power. Qual Health Res. 2016;26(13):1753–60. doi: 10.1177/1049732315617444 26613970

[58] Hennink MM, Kaiser BN, Marconi VC. Code Saturation Versus Meaning Saturation: How Many Interviews Are Enough? Qual Health Res. 2017;27(4):591–608. doi: 10.1177/1049732316665344 27670770 PMC9359070

[59] Ho KHM, Chiang VCL, Leung D. Hermeneutic phenomenological analysis: the “possibility” beyond “actuality” in thematic analysis. J Adv Nurs. 2017;73(7):1757–66. doi: 10.1111/jan.13255 28103404

[60] Salla M. There is no nonviolent future. Social Alternatives. 1996;15(3):41–3.

[61] Burke S. Rethinking ‘validity’ and ‘trustworthiness’ in qualitative inquiry: How might we judge the quality of qualitative research in sport and exercise sciences?. Routledge handbook of qualitative research in sport and exercise. Routledge. 2016:352–62.

[62] Levitt HM, Motulsky SL, Wertz FJ, Morrow SL, Ponterotto JG. Recommendations for designing and reviewing qualitative research in psychology: Promoting methodological integrity. Qualitative Psychology. 2017;4(1):2–22. doi: 10.1037/qup0000082

[63] England DNA philosophy unveiled at St. George’s Park. Available from: https://www.thefa.com/news/2014/dec/04/england-dna-launch. 2014. Accessed 2025 April 20.

[64] Hewitt A, Greenham G, Norton K. Game style in soccer: what is it and can we quantify it? International Journal of Performance Analysis in Sport. 2016;16(1):355–72. doi: 10.1080/24748668.2016.11868892

[65] Gollan S, Ferrar K, Norton K. Characterising game styles in the English Premier League using the “moments of play” framework. International Journal of Performance Analysis in Sport. 2018;18(6):998–1009. doi: 10.1080/24748668.2018.1539383

[66] Barraclough S, Till K, Kerr A, Emmonds S. Methodological Approaches to Talent Identification in Team Sports: A Narrative Review. Sports (Basel). 2022;10(6):81. doi: 10.3390/sports10060081 35736821 PMC9227581

[67] U.S. Soccer unveils strategy with ‘The U.S. Way’ rollout in 2025 as USWNT prepares for January camps. Available from: https://www.cbssports.com/soccer/news/u-s-soccer-unveils-strategy-with-the-u-s-way-rollout-in-2025-as-uswnt-prepares-for-january-camps/. Accessed 2025 April 20.

[68] Andrew M, Baptiste GZ, Reeves MJ, Roberts SJ, McRobert AP, Ford PR. The developmental activities of skilled youth CONCACAF soccer players and the contribution of their development system. International Journal of Sports Science & Coaching. 2021;17(6):1363–77. doi: 10.1177/17479541211061036

[69] Christensen MK. “An Eye for Talent”: Talent Identification and the “Practical Sense” of Top-Level Soccer Coaches. Sociology of Sport Journal. 2009;26(3):365–82. doi: 10.1123/ssj.26.3.365

[70] Den Hartigh RJR, Niessen ASM, Frencken WGP, Meijer RR. Selection procedures in sports: Improving predictions of athletes’ future performance. Eur J Sport Sci. 2018;18(9):1191–8. doi: 10.1080/17461391.2018.1480662 29856681

[71] Thompson CJ, Noon M, Towlson C, Perry J, Coutts AJ, Harper LD, et al. Understanding the presence of mental fatigue in English academy soccer players. J Sports Sci. 2020;38(13):1524–30. doi: 10.1080/02640414.2020.1746597 32212903

[72] Fowler P, Duffield R, Vaile J. Effects of domestic air travel on technical and tactical performance and recovery in soccer. Int J Sports Physiol Perform. 2014;9(3):378–86. doi: 10.1123/IJSPP.2013-0484 24755963

[73] Forcher L, Forcher L, Wäsche H, Jekauc D, Woll A, Altmann S. The influence of tactical formation on physical and technical match performance in male soccer: A systematic review. International Journal of Sports Science & Coaching. 2022;18(5):1820–49. doi: 10.1177/17479541221101363

[74] McGuckin TA, Sinclair WH, Sealey RM, Bowman P. The effects of air travel on performance measures of elite Australian rugby league players. Eur J Sport Sci. 2014;14 Suppl 1:S116–22. doi: 10.1080/17461391.2011.654270 24444195

[75] Lago C, Casais L, Dominguez E, Sampaio J. The effects of situational variables on distance covered at various speeds in elite soccer. European Journal of Sport Science. 2010;10(2):103–9. doi: 10.1080/17461390903273994

[76] Morganti G, Kelly AL, Apollaro G, Pantanella L, Esposito M, Grossi A, et al. All roads lead to Rome? Exploring birthplace effects and the ‘southern question’ in Italian soccer. Soccer & Society. 2023;25(1):62–75. doi: 10.1080/14660970.2023.2226077

[77] Hernández-Simal L, Calleja-González J, Calvo AL, Aurrekoetxea-Casaus M. Birthplace Effect in Soccer: A Systematic Review. J Hum Kinet. 2024;94:227–42. doi: 10.5114/jhk/186935 39563766 PMC11571467

[78] Wiseman AC, Bracken N, Horton S, Weir PL. The Difficulty of Talent Identification: Inconsistency among Coaches through Skill-Based Assessment of Youth Hockey Players. International Journal of Sports Science & Coaching. 2014;9(3):447–55. doi: 10.1260/1747-9541.9.3.447

[79] Leyhr D, Bergmann F, Schreiner R, Mann D, Dugandzic D, Höner O. Relative Age-Related Biases in Objective and Subjective Assessments of Performance in Talented Youth Soccer Players. Front Sports Act Living. 2021;3:664231. doi: 10.3389/fspor.2021.664231 34056590 PMC8160372

[80] Lüdin D, Donath L, Cobley S, Mann D, Romann M. Player-labelling as a solution to overcome maturation selection biases in youth football. J Sports Sci. 2022;40(14):1641–7. doi: 10.1080/02640414.2022.2099077 35969578

[81] Bergkamp TLG, Niessen ASM, den Hartigh RJR, Frencken WGP, Meijer RR. Methodological Issues in Soccer Talent Identification Research. Sports Med. 2019;49(9):1317–35. doi: 10.1007/s40279-019-01113-w 31161402 PMC6684562

[82] Bergkamp TLG, Frencken WGP, Niessen ASM, Meijer RR, den Hartigh RJR. How soccer scouts identify talented players. Eur J Sport Sci. 2022;22(7):994–1004. doi: 10.1080/17461391.2021.1916081 33858300

[83] Larkin P, Marchant D, Syder A, Farrow D. An eye for talent: The recruiters’ role in the Australian Football talent pathway. PLoS One. 2020;15(11):e0241307. doi: 10.1371/journal.pone.0241307 33137113 PMC7605670

[84] Larkin P, O’Connor D. Talent identification and recruitment in youth soccer: Recruiter’s perceptions of the key attributes for player recruitment. PLoS One. 2017;12(4):e0175716. doi: 10.1371/journal.pone.0175716 28419175 PMC5395184

[85] Lund S, Söderström T. To See or Not to See: Talent Identification in the Swedish Football Association. Sociology of Sport Journal. 2017;34(3):248–58. doi: 10.1123/ssj.2016-0144

[86] Barraclough S, Piggott D, Till K, Kerr A, Emmonds S. Creating a shared mental model of performance: Coaches’ perspectives of key position-specific soccer actions. International Journal of Sports Science & Coaching. 2023;19(2):586–603. doi: 10.1177/17479541231205473

[87] Eccles DW, Tenenbaum G. Why an Expert Team Is More than a Team of Experts: A Social-Cognitive Conceptualization of Team Coordination and Communication in Sport. Journal of Sport and Exercise Psychology. 2004;26(4):542–60. doi: 10.1123/jsep.26.4.542

[88] Leyhr D, Bergmann F, Raabe J, Höner O. Multidimensional Performance Assessments in U15 Female Soccer: The Predictive Validity for Different Selection Levels in U17 and Success in Adulthood. Eur J Sport Sci. 2025;25(7):e12335. doi: 10.1002/ejsc.12335 40569648 PMC12199725

[89] McEwan GP, Unnithan VB, Carter M, Dugdale JH, Datson N. Talent identification and development strategies in elite women’s soccer: a pan-European perspective. Sci Med Footb. 2024;1–9. doi: 10.1080/24733938.2024.2404920 39291638

[90] Fuhre J, Øygard A, Sæther SA. Coaches’ Criteria for Talent Identification of Youth Male Soccer Players. Sports (Basel). 2022;10(2):14. doi: 10.3390/sports10020014 35202054 PMC8875243

[91] Lethole L, Kubayi A, Toriola A, Larkin P, Armatas V. Development of the Talent Identification Questionnaire in Soccer for Outfield Players (TIDQ-OP): Coaches’ perceptions of the key attributes for player recruitment. J Sports Sci. 2024;42(4):291–300. doi: 10.1080/02640414.2024.2329432 38477297

[92] Gledhill A, Harwood C. Toward an Understanding of Players’ Perceptions of Talent Development Environments in UK Female Football. Journal of Applied Sport Psychology. 2018;31(1):105–15. doi: 10.1080/10413200.2017.1410254

[93] Hernández-Simal L, Calleja-González J, Larruskain J, Lorenzo Calvo A, Aurrekoetxea-Casaus M. Place Matters: A Study on the Influence of Birthplace and the Place of Development on Soccer Academy Players’ Careers. Sports (Basel). 2024;12(4):99. doi: 10.3390/sports12040099 38668567 PMC11054205

[94] Hancock DJ, Coutinho P, Côté J, Mesquita I. Influences of population size and density on birthplace effects. J Sports Sci. 2018;36(1):33–8. doi: 10.1080/02640414.2016.1276614 28078945

[95] Law MP, Côté J, Ericsson KA. Characteristics of expert development in rhythmic gymnastics: A retrospective study. International Journal of Sport and Exercise Psychology. 2007;5(1):82–103. doi: 10.1080/1612197x.2008.9671814

[96] Till K, Baker J. Challenges and [Possible] Solutions to Optimizing Talent Identification and Development in Sport. Front Psychol. 2020;11:664. doi: 10.3389/fpsyg.2020.00664 32351427 PMC7174680

[97] Painter E, Price M. Creating social capital on soccer fields: immigrant opportunities and gendered barriers in adult soccer leagues. Journal of Ethnic and Migration Studies. 2019;47(7):1631–48. doi: 10.1080/1369183x.2019.1602030

[98] Tompsett J, Knoester C. The Making of a College Athlete: High School Experiences, Socioeconomic Advantages, and the Likelihood of Playing College Sports. Sociology of Sport Journal. 2022;39(2):129–40. doi: 10.1123/ssj.2020-0142

[99] Bowes A, Culvin A, Carrick S. Equal pay debates in international women’s football. Women’s football in a global, professional era. Emerald Publishing Limited. 2023:191–203.

[100] Harrison GE, Vickers E, Fletcher D, Taylor G. Elite female soccer players’ dual career plans and the demands they encounter. Journal of Applied Sport Psychology. 2020;34(1):133–54. doi: 10.1080/10413200.2020.1716871

[101] Cahill G, MacNamara Á. Contextual factors in sport talent identification research. Sport in Society. 2023;27(7):1180–216. doi: 10.1080/17430437.2023.2288149

[102] Gentles JA, Coniglio CL, Besemer MM, Morgan JM, Mahnken MT. The Demands of a Women’s College Soccer Season. Sports (Basel). 2018;6(1):16. doi: 10.3390/sports6010016 29910320 PMC5969200

[103] Dugdale JH, Sanders D, Myers T, Williams AM, Hunter AM. Progression from youth to professional soccer: A longitudinal study of successful and unsuccessful academy graduates. Scand J Med Sci Sports. 2021;31 Suppl 1:73–84. doi: 10.1111/sms.13701 33871087

[104] Soccer Rules of the Game. Available from: https://www.ncaa.org/sports/2013/12/2/soccer-rules-of-the-game.aspx. Accessed 2025 April 10.

[105] Dugdale JH, Sanders D, Myers T, Williams AM, Hunter AM. A case study comparison of objective and subjective evaluation methods of physical qualities in youth soccer players. J Sports Sci. 2020;38(11–12):1304–12. doi: 10.1080/02640414.2020.1766177 32536323

[106] Andrew M, Ford PR, Miller MT, McRobert AP, Foster NC, Seerden G, et al. Bridging the Gap Between Science and Application: The Use of Cocreation Educational Workshops in Professional Youth Soccer. International Sport Coaching Journal. 2022;9(1):82–99. doi: 10.1123/iscj.2020-0054

[107] Howell S, Lee Y, Yi KJ. Nurturing holistic talent, addressing systemic inequity: Canadian coaches’ insights on optimizing youth soccer talent identification and development. International Journal of Sports Science & Coaching. 2024;20(2):443–54. doi: 10.1177/17479541241296454

[108] Grossmann B, Lames M. Relative Age Effect (RAE) in Football Talents – the Role of Youth Academies in Transition to Professional Status in Germany. International Journal of Performance Analysis in Sport. 2013;13(1):120–34. doi: 10.1080/24748668.2013.11868636

